# Preparation and Performance of Phthalocyanine @ Copper Iodide Cluster Nanoparticles for X-Ray-Induced Photodynamic Therapy

**DOI:** 10.3390/molecules30214229

**Published:** 2025-10-29

**Authors:** Wei Xie, Yunan Li, Guoyan Tang, Zhihua Li, Mengyu Yao, Biyuan Zheng, Xingshu Li, Jian-Dong Huang

**Affiliations:** Fujian Provincial Key Laboratory of Cancer Metastasis Chemoprevention and Chemotherapy, College of Chemistry, Fuzhou University, Fuzhou 350108, China; wood316@163.com (W.X.); 231320282@fzu.edu.cn (Y.L.); 15296180832@163.com (G.T.); lizhihua04@163.com (Z.L.); 18597710174@163.com (M.Y.); woshibyz@163.com (B.Z.)

**Keywords:** copper iodide cluster, X-ray-induced photodynamic therapy, phthalocyanine, theranostics, FRET

## Abstract

The efficacy of X-ray-induced photodynamic therapy (X-PDT) for deep tumors is often hindered by conventional scintillators, typically rare-earth nanoparticles plagued by long-term toxicity and suboptimal scintillation yields. Here, we introduce a copper iodide (Cu-I) cluster, Cu_2_I_2_(PPh_3_)_2_(pz), composed of earth-abundant elements, as an efficient and biocompatible energy transducer for X-PDT. A theranostic nanoplatform, CuI@PcNP, was engineered by co-encapsulating the Cu-I cluster and a phthalocyanine photosensitizer (Pc4OH) within a 1,2-distearoyl-sn-glycero-3-phosphoethanolamine-polyethylene glycol-2000 (DSPE-PEG2K) matrix, which confers excellent physiological stability. This nano-architecture ensures nanoscale proximity between the cluster (donor) and photosensitizer (acceptor), facilitating efficient (58%) Förster resonance energy transfer (FRET) while overcoming aggregation-induced quenching. Upon X-ray irradiation, the platform effectively converted X-rays to visible light, activating Pc4OH to generate potent reactive oxygen species (ROS) and inducing significant dose-dependent cytotoxicity in human hepatocellular carcinoma (HepG2) cells. In a murine hepatoma model, enabling image-guided X-PDT that resulted in a 77.4% tumor inhibition rate with negligible systemic toxicity. Collectively, this work pioneers the integration of phthalocyanine with Cu-I clusters, providing a stable and versatile nanoplatform for image-guided X-PDT.

## 1. Introduction

Advanced luminescent materials are pivotal drivers of innovation in nanotechnology and biomedicine [1,2]. While traditional materials like organic dyes and semiconductor quantum dots have baeen instrumental, they often face limitations such as photobleaching and potential heavy-metal toxicity [3,4,5]. In this context, copper iodide (Cu-I) clusters have recently emerged as a compelling class of materials, distinguished by their high photoluminescence quantum yields, often facilitated by efficient thermally activated delayed fluorescence (TADF) mechanisms [6,7,8]. Their remarkable structural diversity allows for precise tuning of optoelectronic properties through ligand engineering, and their composition from earth-abundant elements, including the essential trace element copper, offers a potential advantage in biocompatibility over materials containing toxic heavy metals. These attributes have enabled their successful deployment in solid-state lighting, organic light-emitting diodes (OLEDs), and sensing, positioning them as highly versatile building blocks for next-generation optoelectronic and biomedical applications [7,9,10,11].

Capitalizing on these exceptional luminescent properties, Cu-I clusters offer significant potential as scintillators [12,13]. These are materials capable of absorbing high-energy radiation, such as clinically relevant X-rays, and converting them into lower-energy photons, typically in the visible or near-infrared spectrum. This energy down-conversion is pivotal for overcoming the primary drawback of conventional photodynamic therapy (PDT), a clinically approved treatment that uses a photosensitizer, light, and oxygen to kill cancer cells [14,15]. The therapeutic efficacy of PDT is severely limited by the shallow tissue penetration of light, which is typically less than a centimeter, thereby restricting its application to superficial tumors [16,17].

To overcome this depth limitation, X-ray-induced photodynamic therapy (X-PDT) has emerged as an advanced modality that utilizes deeply penetrating X-rays as an external energy source [18,19]. In an X-PDT system, scintillators absorb the X-ray energy and convert it into visible light in situ, which subsequently activates a co-localized photosensitizer to initiate the photodynamic process in deep-seated tumors [20,21,22]. While rare-earth-based nanoscintillators have been explored for this purpose, the search for alternatives with superior biocompatibility and simpler synthesis continues. The presence of high-atomic-number elements (iodine, Z = 53; copper, Z = 29) within Cu-I clusters enhances their X-ray absorption cross-section, making them intrinsically suited and highly promising candidates for this critical energy transduction role [7,23].

While Cu-I clusters are promising as energy transducers, their utility as scintillators for X-PDT has only recently been explored in a few pioneering studies. Our group was the first to report the application of Cu-I clusters in X-PDT, utilizing Rose Bengal with folic acid targeting to achieve significant tumor inhibition [24]. Shortly thereafter, Chen’s group reported on biodegradable Cu-I@BSA nanoparticles that leverage ion release for ultralow-dose therapy [10]. These initial reports highlight the novelty and potential of this emerging field. Therefore, designing a nanoplatform that co-localizes the scintillator and a suitable photosensitizer to ensure this energy transfer is paramount. Phthalocyanines (Pcs) represent an ideal class of photosensitizers, exhibiting strong Q-band absorption in the phototherapeutic window (600–800 nm) and high singlet oxygen quantum yields [25,26]. Crucially, their intrinsic fluorescence also allows them to function as imaging probes, enabling the development of theranostic agents that combine therapy with real-time diagnosis or tracking. The efficacy of such a system hinges on efficient energy transfer, typically via FRET, from the scintillator to the photosensitizer. This necessitates not only excellent spectral overlap between the scintillator’s emission and the Pc’s absorption but also their co-localization within nanometer proximity (<10 nm), a requirement effectively met by encapsulating both components within a stable, biocompatible nanocarrier [27,28].

To address this multifaceted challenge, this work describes the design and fabrication of a fully integrated theranostic nanoplatform, denoted CuI@PcNP, for image-guided X-PDT. Among various Cu-I clusters, Cu_2_I_2_(PPh_3_)_2_(pz) was selected due to its intense emission centered at ~630 nm, which provides excellent spectral overlap with the Q-band of phthalocyanines, and its favorable solubility for nanoprecipitation. This system was engineered via a facile co-encapsulation of this custom-synthesized Cu-I cluster scintillator and the multifunctional phthalocyanine derivative, Pc4OH, within a clinically relevant 1,2-distearoyl-sn-glycero-3-phosphoethanolamine-polyethylene glycol-2000 (DSPE-PEG2K) matrix, a lipid component of several FDA-approved nanomedicines. This strategy ensures colloidal stability and co-localizes the components for optimal FRET. The study systematically investigates the platform’s photophysical characteristics and colloidal stability, demonstrates X-ray-triggered energy transfer and subsequent reactive oxygen species (ROS) generation, and evaluates its comprehensive in vitro and in vivo theranostic performance—including fluorescence imaging-guided biodistribution and potent tumor inhibition. This work not only validates the feasibility of using Cu-I clusters as efficient and biocompatible energy transducers in X-PDT but also pioneers a new strategy for applying this unique class of materials in the development of advanced, image-guided cancer therapeutics.

## 2. Results and Discussion

### 2.1. Synthesis and Characterization

#### 2.1.1. Synthesis of Copper Iodide Clusters and Phthalocyanines

The copper iodide cluster complex Cu_2_I_2_(PPh_3_)_2_(pz) was successfully prepared by a strategic ligand exchange method and subsequently characterized [29,30]. The direct synthesis of complexes between copper(I) iodide and pyridine-type ligands often results in mixed phases, including 0D-Cu_4_I_4_(L)_4_ tetramers, 1D-CuI(L) ladder-like chain structures, and 0D-Cu_2_I_2_(L)_4_ dimers. This heterogeneity makes it challenging to isolate the target dimeric structure, which is typically responsible for the high emission intensity and quantum yield. To circumvent this issue, we adopted a ligand exchange strategy that employs a pre-formed 0D-Cu_2_I_2_(L)_4_ dimer as a molecular precursor [10,31]. This approach was designed to preserve the integrity of the dimeric Cu_2_I_2_ core, thereby ensuring the desired strong emissive properties of the final product.

Specifically, the intermediate Cu_2_I_2_(3-mpy)_4_ was first prepared by reacting CuI with 3-methylpyridine (3-mpy). Given that the Cu–N coordination bond in the pyridine complex is relatively labile and susceptible to ligand exchange, a selective substitution was subsequently performed on Cu_2_I_2_(3-mpy)_4_ using triphenylphosphine (PPh_3_) to yield Cu_2_I_2_(PPh_3_)_2_(3-mpy)_2_. In this step, PPh_3_, as a strongly electron-donating and sterically bulky ligand, contributes to the formation of a more stable copper iodide cluster. Finally, the target product, Cu_2_I_2_(PPh_3_)_2_(pz), was obtained through a final ligand exchange with an excess of pyrazine (pz). This controlled, stepwise ligand exchange strategy not only effectively prevents the formation of undesirable phases and ensures the structural stability of the copper iodide core but also enables precise modulation of the physicochemical properties of the hybrid compound by introducing ligands with distinct steric profiles and solubility characteristics. This lays a solid foundation for the subsequent investigation of its optical emission properties. Its structure is shown in Figure S1. The phase composition of the synthesized copper iodide cluster was confirmed by Powder X-ray diffraction (PXRD), and its PXRD pattern (Figure S2) was consistent with previously reported findings [29,30].

The target phthalocyanine derivative, Pc4OH, was prepared following a reported literature procedure [32]. The synthesis route involved two steps: first, under the catalysis of potassium carbonate (K_2_CO_3_), a nucleophilic aromatic substitution reaction between one terminal hydroxyl group of tetraethylene glycol and 3-nitrophthalonitrile afforded the key intermediate, PTOH. Subsequently, a cyclization reaction was carried out with the intermediate PTOH, phthalonitrile, zinc acetate (Zn(OAc)_2_), and 1,8-diazabicyclo [5.4.0]undec-7-ene (DBU) in anhydrous n-pentanol to yield the final product, Pc4OH (Scheme S1). PTOH and Pc4OH were characterized by ^1^H NMR and high-resolution mass spectrometry (HRMS), with the corresponding data presented in the Figures S3–S6.

#### 2.1.2. Characterization of Copper Iodide Clusters and Phthalocyanines

The photophysical properties of the synthesized copper iodide cluster were first investigated. The X-ray excited luminescence (XEL) spectrum of Cu_2_I_2_(PPh_3_)_2_(pz) was measured in the solid state at room temperature. As shown in Figure S7a, under X-ray irradiation, the cluster displayed a strong, broad emission band with an emission maximum at 630 nm. To establish the validity of using a more accessible excitation source for subsequent studies, we also measured its photoluminescence (PL) spectrum under 360 nm excitation. Encouragingly, the resulting photoluminescence spectrum (Figure S7b showed a nearly identical emission profile and maximum, confirming that the emissive pathway of the cluster is independent of the high-energy excitation mode, consistent with Kasha’s rule [33]. Crucially, this emission spectrum exhibits significant spectral overlap with the characteristic Q-band absorption of the photosensitizer, Pc4OH (Figure S8). This excellent spectral alignment is a fundamental prerequisite for efficient FRET from the scintillating cluster (the energy donor) to Pc4OH (the energy acceptor). This property validates the potential for constructing an efficient X-ray-induced photodynamic therapy system by co-encapsulating these two components within a single nanoplatform.

In addition to its optical properties, the physicochemical characteristics relevant for nanoparticle fabrication were assessed. The Cu_2_I_2_(PPh_3_)_2_(pz) cluster demonstrated excellent solubility in dimethyl sulfoxide (DMSO). This high solubility is a critical attribute that enables the successful fabrication of the nanoplatform via the nanoprecipitation method.

#### 2.1.3. Preparation and Characterization of CuI@PcNP

**Scheme 1:**
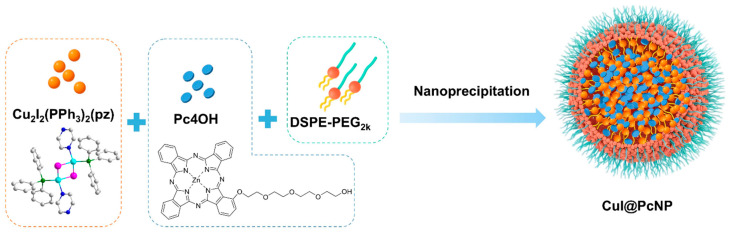
Fabrication of CuI@PcNP via nanoprecipitation. The process involves co-precipitation of the scintillator copper iodide cluster (Cu_2_I_2_(PPh_3_)_2_(pz), PPh_3_ = triphenylphosphine, pz = pyrazine) and the photosensitizer phthalocyanine (Pc4OH) with the amphiphilic lipid DSPE-PEG2K, resulting in a stable nanoparticle featuring a hydrophobic core and a hydrophilic PEG corona.

**Figure 1:**
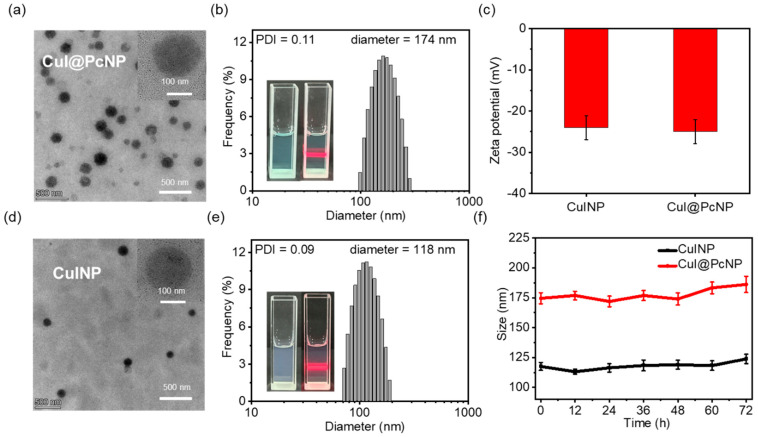
Characterization of nanoparticles. (**a**) Transmission electron microscopy (TEM) image of CuI@PcNP. (**b**) Hydrodynamic size distribution of CuI@PcNP determined by dynamic light scattering (DLS). The inset shows a photograph demonstrating the Tyndall effect. (**c**) Zeta potentials of CuINP and CuI@PcNP. (**d**) TEM image of CuINP. (**e**) Hydrodynamic size distribution of CuINP. (**f**) Colloidal stability of CuINP and CuI@PcNP in aqueous solution over a period of up to 72 h.

The theranostic nanoparticle designated CuI@PcNP was fabricated using a facile and reproducible nanoprecipitation method (Scheme 1). Briefly, a DMSO solution containing the hydrophobic copper cluster (Cu_2_I_2_(PPh_3_)_2_(pz)), the photosensitizer (Pc4OH), and the amphiphilic lipid (DSPE-PEG2K) was added dropwise into an aqueous phase under vigorous stirring. This rapid solvent displacement induced the co-precipitation of the water-insoluble components, while the DSPE-PEG2K molecules self-assembled at the interface, encapsulating the hydrophobic cargo to form stable nanoparticles, where the hydrophobic components form a core encapsulated by a hydrophilic PEG corona. The physicochemical properties of the as-prepared nanoparticles were then systematically characterized. Transmission electron microscopy (TEM) images revealed that the CuI@PcNP possessed a uniform spherical morphology with excellent dispersity (Figure 1a). Further analysis by dynamic light scattering (DLS) determined the hydrodynamic diameter to be approximately 174 nm with a narrow polydispersity index (PDI) of 0.11, indicating high homogeneity (Figure 1b). The successful formation of a stable colloidal dispersion was further confirmed visually by a distinct Tyndall effect when the solution was irradiated with a laser beam (Figure 1b, inset). Furthermore, the zeta potential was measured to be −25.0 mV (Figure 1c). This negative surface charge, attributed to the phosphate groups in DSPE-PEG2K, provides strong electrostatic repulsion between particles, thereby contributing to their outstanding colloidal stability.

For comparative analysis, a control nanoparticle, CuINP (encapsulating only the copper cluster), and a control formulation, PcNP (encapsulating only the photosensitizer), were prepared using an identical protocol. The CuINP nanoparticles were observed by TEM to possess a spherical morphology with uniform size distribution (Figure 1d), and their hydrodynamic diameter and PDI were determined to be 118 nm and 0.09, respectively (Figure 1e). In contrast, while the PcNP formed a colloidal suspension as confirmed by a distinct Tyndall effect and a negative zeta potential (Figure S9), DLS analysis revealed the formation of highly heterogeneous aggregates (PDI = 2.15). This result confirms its suitability as a control representing the photosensitizer alone in solution. The colloidal stability of the successfully formed CuI@PcNP and CuINP was subsequently evaluated over three days in an aqueous solution. As demonstrated in Figure 1f, Figures S10 and S11, the nanoparticles exhibited negligible changes in hydrodynamic size, UV-vis absorption, and fluorescence intensity, which confirms their excellent stability for subsequent biological applications.

**Figure 2:**
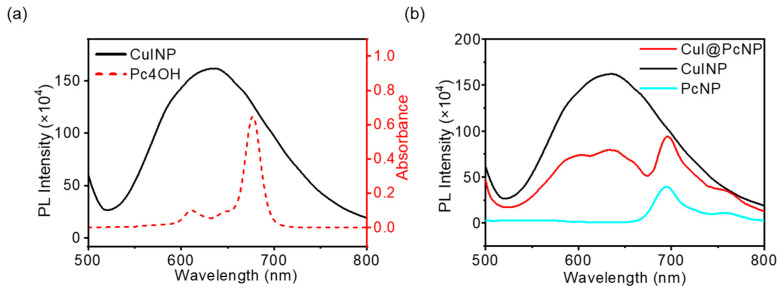
Spectroscopic evidence for the feasibility of Förster resonance energy transfer (FRET) within CuI@PcNP. (**a**) The photoluminescence (PL) spectrum of CuINP (donor) in water, excited at 475 nm, shown alongside the UV-vis absorption spectrum of Pc4OH (acceptor). The spectral overlap is a prerequisite for FRET. (**b**) PL spectra of CuI@PcNP, CuINP, and PcNP in water upon excitation at 475 nm. The concentrations were equivalent to 0.5 mg/mL of the Cu-I cluster and/or 5 μmol/L of Pc4OH.

The successful co-encapsulation of the scintillator and photosensitizer set the stage to verify the key energy transfer process. While the ultimate therapeutic application relies on X-ray excitation, the XEL signal from the dilute CuINP aqueous colloidal dispersion was below the instrumental detection limit. However, based on our finding that the emission profile of the solid-state cluster is independent of the excitation mode (X-ray vs. UV-vis), 475 nm light was therefore utilized as the excitation source to probe the photophysical events within the nanoparticles. To investigate the possibility of FRET from the CuINP (donor) to the encapsulated Pc4OH (acceptor), the spectral properties of the components were first examined. As shown in Figure 2a, the fluorescence emission spectrum of the donor, CuINP, excited at 475 nm, exhibits a strong peak centered at 635 nm. This emission band shows excellent spectral overlap with the characteristic Q-band absorption of the acceptor, Pc4OH. This significant overlap fulfills the fundamental prerequisite for an efficient FRET process.

The occurrence of FRET was then unequivocally confirmed by comparing the fluorescence spectra of the nanoparticles, as depicted in Figure 2b. Upon excitation at 475 nm, the donor-only CuINP solution displayed the strong emission at 635 nm. In stark contrast, the CuI@PcNP colloidal dispersion (at concentrations equivalent to 0.5 mg/mL of the Cu-I cluster and 5 μmol/L of Pc4OH) showed a significant attenuation of this donor emission and, concurrently, a fluorescence emission peak from the phthalocyanine acceptor at 696 nm, which was dramatically enhanced compared to the weak intrinsic fluorescence of the acceptor-only PcNP control. This simultaneous donor fluorescence attenuation and acceptor fluorescence enhancement is the definitive signature of FRET from the copper cluster to the phthalocyanine. The FRET efficiency (E) was calculated to be 58% based on the quenching of the donor’s fluorescence. The efficiency was determined using the formula E = 1 − (F_DA_/F_D_), where F_D_ is the fluorescence intensity of the donor alone and F_DA_ is the intensity of the donor in the presence of the acceptor. To ensure accuracy, the donor was selectively excited at 475 nm, and its emission intensity was measured at its peak of 635 nm. At this wavelength, the fluorescence emission from the Pc4OH acceptor is negligible, which allows for an accurate determination of the donor’s fluorescence intensity without requiring significant spectral correction.

**Figure 3:**
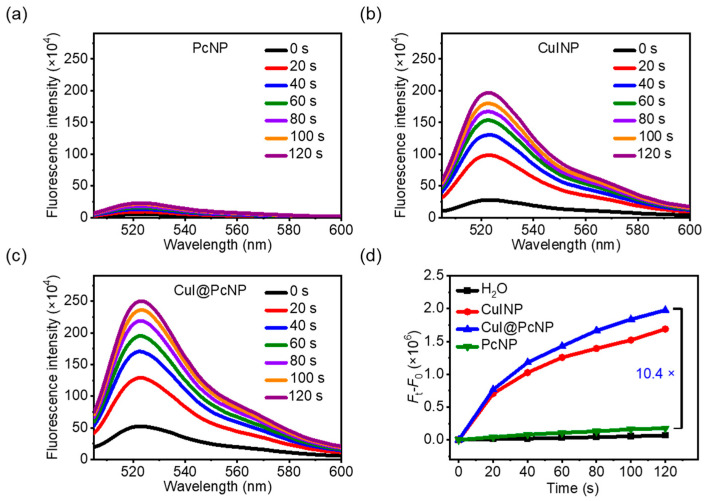
Evaluation of X-ray-induced reactive oxygen species (ROS) generation, detected using the DCFH-DA probe. (**a**–**c**) Time-dependent fluorescence emission spectra of DCF (λ_ex_ = 488 nm) in the presence of (**a**) PcNP, (**b**) CuINP, and (**c**) CuI@PcNP under continuous X-ray irradiation. (**d**) Corresponding plot of DCF fluorescence intensity enhancement at 525 nm versus irradiation time, comparing the ROS generation efficiency of different nanoparticles and a water control. For all nanoparticle groups, the concentrations of the active components were maintained at 0.5 mg/mL for Cu_2_I_2_(PPh_3_)_2_(pz) and/or 5 μmol/L for Pc4OH.

Finally, the ultimate therapeutic function—X-ray-induced ROS generation—was quantified using 2′,7′-dichlorodihydrofluorescein diacetate (DCFH-DA) as a fluorescent probe. DCFH-DA is hydrolyzed to non-fluorescent DCFH, which is then oxidized by ROS to the highly fluorescent 2′,7′-dichlorofluorescein (DCF). As shown in Figure 3, upon exposure to X-ray irradiation, the CuI@PcNP solution induced a marked, time-dependent increase in the DCF fluorescence intensity at 525 nm. Interestingly, the CuINP group also produced a substantial amount of ROS, indicating that the copper iodide cluster itself possesses intrinsic radiosensitizing capabilities. Nevertheless, the CuI@PcNP group consistently generated a significantly higher level of ROS, with the amount produced being 1.2-fold and 10.4-fold that of the CuINP and PcNP groups, respectively. This result powerfully demonstrates a synergistic effect. While the cluster itself is active, the FRET-mediated activation of Pc4OH provides a more potent, additional pathway for ROS production, thus validating the rational design of our integrated X-PDT nanoplatform.

### 2.2. In Vitro X-PDT Efficacy

**Figure 4:**
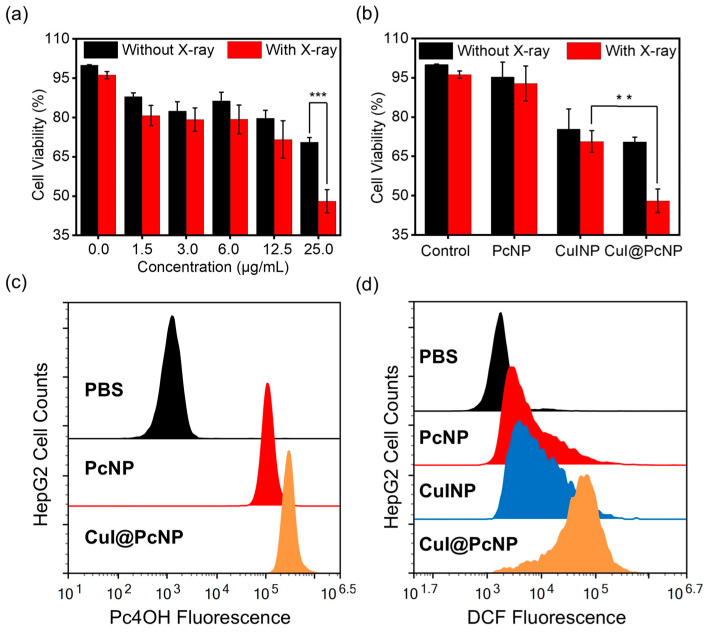
In vitro evaluation of X-ray-induced photodynamic therapy (X-PDT) efficacy and its mechanism in HepG2 cells. (**a**) Viability of HepG2 cells incubated with varying concentrations of CuI@PcNP, with or without X-ray irradiation (1.92 Gy), as measured by MTT assay. (**b**) Comparative cytotoxicity of different nanoparticles (PcNP, CuINP, and CuI@PcNP) in HepG2 cells under the same irradiation conditions. (**c**) Cellular uptake of nanoparticles analyzed by flow cytometry after a 4 h incubation. (**d**) Intracellular ROS generation in HepG2 cells under various treatments with X-ray irradiation, detected using a DCFH-DA probe. For panels (**b**–**d**), nanoparticles were used at concentrations equivalent to 25 μg/mL of the Cu-I cluster (for CuINP and CuI@PcNP) and 5 μmol/L of Pc4OH (for PcNP). Data in (**a**,**b**) are presented as mean ± SD (*n* = 3). ** *p* < 0.01, *** *p* < 0.001.

The in vitro X-PDT efficacy of CuI@PcNP and PcNP was evaluated against human hepatocellular carcinoma (HepG2) cells using a standard 3-(4,5-dimethylthiazol-2-yl)-2,5-diphenyltetrazolium bromide (MTT) assay [34]. First, the dose-dependent cytotoxicity of CuI@PcNP was assessed at an X-ray dose of 1.92 Gy (40 kV, 80 μA, consistent with low-dose standards in X-PDT literature, e.g., 2–4 Gy for efficacy [10,19]). As shown in Figure 4a, the nanoparticles exhibited mild dark toxicity (without X-ray). In contrast, upon exposure to X-rays (40 kV, 80 μA, 1.92 Gy), CuI@PcNP induced a pronounced, dose-dependent decrease in cell viability. This demonstrates a clear X-ray-activated therapeutic effect.

To elucidate the contributions of each component, a comparative study was performed at a fixed nanoparticle concentration (25 μg/mL of the Cu-I cluster, equivalent to approx. 25.4 µmol/L) (Figure 4b). Control experiments established that X-ray irradiation alone did not significantly impact cell viability. Under X-ray irradiation, the CuI@PcNP group exhibited potent cytotoxicity, reducing cell viability to 48.0 ± 4.5%, which was significantly lower than all other groups (** *p* < 0.01). The CuINP group also displayed moderate cytotoxicity (70.7 ± 8.2% viability), confirming the intrinsic radiosensitizing property of the copper iodide cluster. In contrast, the PcNP group showed negligible therapeutic efficacy (92.8 ± 6.7% viability). These results powerfully confirm that the integrated CuI@PcNP nanoplatform achieves a superior therapeutic outcome via a synergistic effect between its components.

To investigate the cellular mechanisms underlying these differential cytotoxic outcomes, we first assessed the cellular uptake using flow cytometry. As shown in Figure 4c, after a 4 h incubation, both PcNP and CuI@PcNP exhibited strong fluorescence signals within HepG2 cells compared to the PBS control, indicating that both formulations types were efficiently internalized.

Subsequently, the intracellular ROS generation was quantified using the DCFH-DA probe. The results, depicted in Figure 4d, perfectly mirrored the cytotoxicity data. Upon X-ray irradiation, the CuI@PcNP-treated cells displayed the strongest fluorescence signal, signifying the highest level of ROS production. The CuINP group also generated a notable amount of ROS, consistent with its moderate cytotoxicity. Conversely, the PcNP group produced minimal ROS, explaining its lack of X-PDT efficacy. Collectively, these mechanistic studies demonstrate that the superior therapeutic efficacy of CuI@PcNP is directly attributable to its enhanced ability to generate cytotoxic ROS within cancer cells, a process enabled by the efficient FRET from the scintillating core to the photosensitizer upon X-ray activation.

### 2.3. In Vivo Biodistribution and Antitumor Efficacy

#### 2.3.1. In Vivo Fluorescence Imaging and Tumor Accumulation

**Figure 5:**
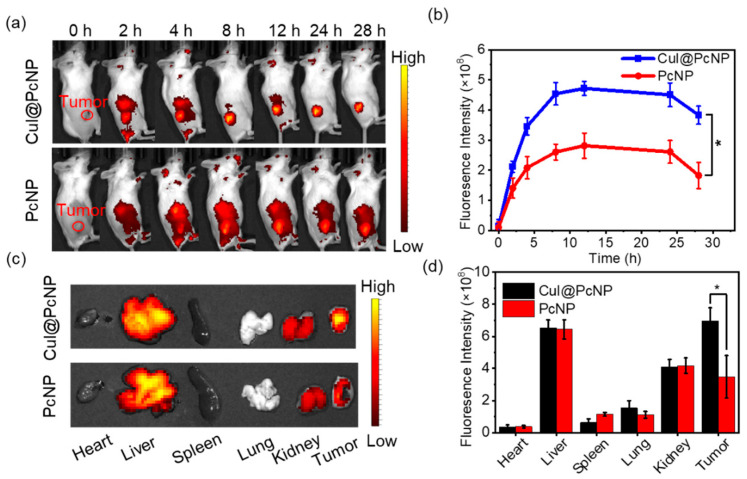
In vivo biodistribution and tumor accumulation of nanoparticles in H22 tumor-bearing mice. (**a**) Time-dependent in vivo fluorescence imaging of mice after intravenous injection of CuI@PcNP or PcNP. (**b**) Quantitative analysis of the fluorescence intensity at the tumor site over 28 h. (**c**) Ex vivo fluorescence images of major organs and tumors harvested 28 h post-injection. (**d**) Corresponding semi-quantitative analysis of the fluorescence signals in the dissected organs and tumors. All fluorescence images were acquired using an IVIS system (λ_ex_ = 605 nm, λ_em_ = 680 nm). Data are presented as mean ± SD (*n* = 3). * *p* < 0.05.

Inspired by the promising in vitro results, we proceeded to investigate the biodistribution of CuI@PcNP and the PcNP control in vivo using an H22 murine hepatoma model. As visualized in the real-time fluorescence images (Figure 5a), CuI@PcNP demonstrated rapid and preferential accumulation at the tumor site following intravenous injection. Quantitative analysis of the tumor fluorescence intensity (Figure 5b) revealed that the signal emerged as early as 2 h post-injection, progressively intensified, and reached its peak at 12 h, which was consequently selected as the optimal time point for subsequent X-ray therapy. In contrast, the control PcNP group exhibited significantly weaker and more diffuse fluorescence signals, resulting in substantially lower tumor accumulation at all time points (* p < 0.05).

To further validate these biodistribution patterns, major organs and tumors were harvested for ex vivo imaging 28 h post-injection. The images and corresponding quantification (Figure 5c,d) clearly showed that CuI@PcNP was predominantly retained in the tumor and liver. Notably, the fluorescence intensity in the tumor was markedly higher than in other major organs such as the kidneys and spleen. Conversely, PcNP displayed poor tumor targeting, with higher accumulation observed in the liver and kidneys than in the tumor itself. These results collectively confirm that the CuI@PcNP nanostructure facilitates preferential accumulation at the tumor site, which is attributed to the enhanced permeability and retention (EPR) effect.

#### 2.3.2. In Vivo X-PDT Efficacy

**Figure 6:**
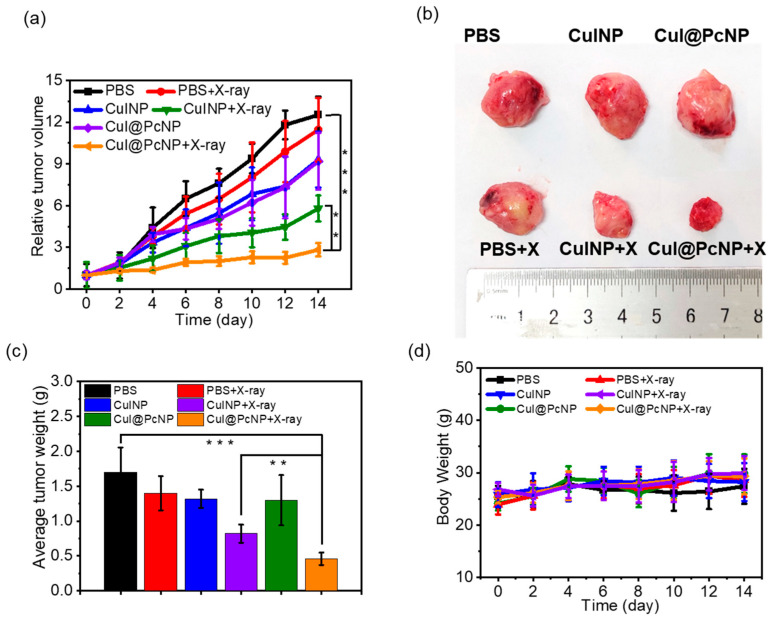
In vivo evaluation of X-PDT and intrinsic antitumor effects in H22 tumor-bearing mice. (**a**) Relative tumor volume growth curves over 14 days for different treatment groups. (**b**) Representative photographs of tumors excised from each group on day 14 post-treatment. In the figure, X denotes X-ray. (**c**) Average weights of the excised tumors. (**d**) Body weight changes in the mice throughout the treatment period, indicating negligible systemic toxicity. For X-ray irradiation, a 5 cm thick pork tissue was placed over the tumor to simulate a deep-seated tumor model. Data are presented as mean ± SD (*n* = 5). ** *p* < 0.01, *** *p* < 0.001.

Leveraging the optimized 12 h accumulation window, we evaluated the in vivo antitumor efficacy. To simulate a more clinically relevant scenario for deep-seated tumors, a 5 cm thick layer of pork tissue was used to attenuate the X-ray beam before it reached the tumor. The therapeutic outcomes were monitored over 14 days. The X-ray dose employed (40 kV, 80 μA, 8 min) aligns with low-dose X-PDT standards in the literature for in vivo studies (e.g., 1–10 Gy achieving efficacy with minimal toxicity [10,19]), enabling deep penetration while minimizing side effects. As depicted by the tumor growth curves, final tumor images, and weights (Figure 6a–c), the CuI@PcNP + X-ray group demonstrated outstanding therapeutic efficacy, achieving a tumor inhibition rate of 77.4% and resulting in significant tumor reduction. The CuINP + X-ray group also displayed considerable tumor suppression (53.8% inhibition), confirming the intrinsic radiosensitizing effect of the copper iodide core. In contrast, tumors in the PBS and PBS + X-ray control groups grew rapidly. Throughout the treatment period, no significant body weight fluctuations were observed in any group (Figure 6d), indicating the excellent biocompatibility and minimal systemic toxicity of the nanotherapeutics.

## 3. Materials and Methods

### 3.1. Materials and Instruments

All chemicals and reagents were of analytical grade and used as received without further purification. 3-Methylpyridine (3-mpy, 99%) and 3-(4,5-dimethylthiazol-2-yl)-2,5-diphenyltetrazolium bromide (MTT, 98%) were purchased from Sigma-Aldrich Reagent Co., Ltd. (St. Louis, MO, USA). Cuprous iodide (CuI, 99.5%), triphenylphosphine (PPh_3_, 98%), and pyrazine (pz, 99%) were obtained from Beijing Innochem Science & Technology Co., Ltd. (Beijing, China). Tetraethylene glycol (99%) was acquired from Alfa Chemical Co., Ltd. (Zhengzhou, China). 3-Nitrophthalonitrile (99%) was from Adamas Reagent Ltd. (Shanghai, China), and 1,8-Diazabicyclo [5.4.0]undec-7-ene (DBU, 98%) was from Tokyo Chemical Industry Co., Ltd. (Tokyo, Japan). All organic solvents, including DMSO, acetone, N,N-dimethylformamide (DMF), dichloromethane (DCM), n-pentanol, and ethyl acetate (EA), all ≥99% purity, were supplied by China National Pharmaceutical Group Co., Ltd. (Beijing, China).

Hydrodynamic size distributions of the nanoparticles were determined by dynamic light scattering (DLS) on an Anton Paar Litesizer 500 (Anton Paar, Graz, Austria). Transmission electron microscopy (TEM) images were acquired with a TECNAI G2 F20 microscope (Thermo Fisher Scientific, Waltham, MA, USA) operating at an accelerating voltage of 200 kV. Powder X-ray powder diffraction (PXRD) patterns were recorded on a Rigaku Ultima IV diffractometer (Rigaku, Tokyo, Japan) using Co Kα radiation (λ = 1.790 Å) over a 2θ range of 5–50°. XEL spectra were recorded on an Edinburgh Instruments FS5 spectrofluorometer (Edinburgh Instruments, Livingston, UK) equipped with a Mini-X X-ray tube source (Amptek, Bedford, MA, USA). A Moxtek^®^ MAGPRO X-ray source (Moxtek, Orem, UT, USA) was utilized for all irradiation procedures in both the in vitro and in vivo experiments. The dose rate of the X-ray tube was calibrated using a RaySafe X2 R/F Si dosimeter (Fluke Biomedical, Everett, WA, USA). UV–vis absorption spectra were measured using a Shimadzu UV-2450 spectrophotometer (Shimadzu, Kyoto, Japan).

All quantitative data are presented as mean ± standard deviation (SD). Statistical significance for comparisons between two groups was determined using a two-tailed Student’s *t*-test. For comparisons involving more than two groups, one-way analysis of variance (ANOVA) was performed, followed by pairwise comparisons using Student’s *t*-test. Analyses were conducted using SPSS software (version 19.0). *p*-values were considered statistically significant as follows: * p < 0.05, ** p < 0.01, and *** p < 0.001.

### 3.2. Synthesis of Copper Iodide Clusters

The synthesis of the target copper iodide cluster was performed via a multi-step ligand-exchange strategy, adapted from a previously reported method [29,30].

#### 3.2.1. Synthesis of Precursor Cu_2_I_2_(3-mpy)_4_

In a 50 mL flask, CuI (0.19 g, 1.0 mmol) was dispersed in acetone (10 mL). To this suspension, 3-methylpyridine (3-mpy, 0.37 g, 4.0 mmol) was added dropwise under continuous magnetic stirring at room temperature. A white precipitate formed immediately. After stirring for 10 min, the product was collected by filtration, washed with a small amount of cold acetone, and dried under vacuum. This precursor was prepared in bulk for subsequent reactions. (Yield: 81% based on Cu).

#### 3.2.2. Synthesis of Precursor Cu_2_I_2_(PPh_3_)_2_(3-mpy)_2_

The precursor Cu_2_I_2_(3-mpy)_4_ (0.15 g, 0.2 mmol) was dissolved in acetone (10 mL). Separately, triphenylphosphine (PPh_3_, 0.13 g, 0.5 mmol) was dissolved in toluene (2 mL). The PPh_3_ solution was then added to the acetone solution under magnetic stirring. A precipitate formed within minutes. The reaction was allowed to proceed for 2 h before the solid product was collected by filtration, washed with acetone, and dried. (Yield: 75% based on Cu).

#### 3.2.3. Synthesis of Final Cluster Cu_2_I_2_(PPh_3_)_2_(pz)

This final ligand-exchange step was crucial for obtaining the target cluster. The precursor Cu_2_I_2_(PPh_3_)_2_(3-mpy)_2_ (0.11 g, 0.1 mmol) and an excess of pyrazine (pz, 0.04 g, 0.5 mmol) were added to toluene (10 mL) in a sealed reaction vial. The mixture was heated to 80 °C and stirred for 24 h. After cooling to room temperature, the resulting yellow crystalline powder was collected by filtration. To ensure purity, the product was washed sequentially with toluene and diethyl ether to remove any unreacted reagents and byproducts, and finally dried overnight in a vacuum oven. (Yield: 60% based on Cu).

### 3.3. Synthesis of Phthalocyanines

#### 3.3.1. Synthesis of Precursor PTOH

To a solution of tetraethylene glycol (5.83 g, 30 mmol) and 3-nitrophthalonitrile (0.87 g, 5 mmol) in anhydrous DMF (20 mL), anhydrous K_2_CO_3_ (4.78 g, 30 mmol) was added in one portion. The reaction mixture was stirred at 30 °C for 24 h. Upon completion, the solvent was removed under reduced pressure. The resulting residue was partitioned between deionized water and dichloromethane (DCM). The organic layer was collected, dried over anhydrous MgSO_4_, filtered, and concentrated by rotary evaporation to yield the pure product as a viscous oil. (1.1 g, 70%). ^1^H NMR (500 MHz, DMSO-*d*_6_, ppm): δ 7.86 (t, J = 8.4 Hz, 1H), 7.68 (t, J = 7.6 Hz, 2H), 4.57 (t, J = 5.4 Hz, 1H), 4.37 (t, J = 4.5, 2H), 3.80 (t, J = 4.5, 2H), 3.61 (t, J = 5.7, 2H), 3.53–3.45 (m, 8H), 3.39 (t, J = 5.5, 2H). HRMS (ESI): m/z calcd for C_16_H_20_N_2_O_5_ [M+H]^+^, 321.1445; found, 321.1441.

#### 3.3.2. Synthesis of Phthalocyanines Derivatives (Pc4OH)

A mixture of the precursor PTOH (0.30 g, 1.0 mmol) and phthalonitrile (0.64 g, 5.0 mmol) was dissolved in n-pentanol (35 mL) under a nitrogen atmosphere. The solution was heated to 90 °C until all solids dissolved completely. Anhydrous zinc acetate (0.30 g, 1.7 mmol) and DBU (0.4 mL) were then added sequentially. The reaction temperature was raised to 135 °C and maintained for 12 h with vigorous stirring. After cooling to room temperature, the solvent was removed in vacuo. The crude residue was first purified by silica gel column chromatography (eluent: ethyl acetate/DCM). The collected fractions containing the product were combined and further purified by gel permeation size-exclusion chromatography (GPC; Bio-Rad Bio-Beads S-X3 column (Bio-Rad Laboratories, Hercules, CA, USA), eluent: DMF) to afford the final compound Pc4OH as a dark green solid. (46.15 mg, 6%). ^1^H NMR (500 MHz, DMSO-*d*_6_, ppm): δ 9.38–9.32 (m, 6H), 9.26 (dd, J = 3.2 Hz, 7.8 Hz, 1H), 8.93 (d, J = 7.5 Hz, 1H), 8.25–8.23 (m, 5H), 8.10 (t, J = 7.7 Hz, 1H), 7.72 (d, J = 8.1 Hz, 1H), 4.87 (t, J = 5.5 Hz, 2H), 4.51 (t, J = 5.5 Hz, 1H), 4.40 (t, J = 4.6 Hz, 2H), 4.08 (t, J = 4.9 Hz, 2H), 3.75 (t, J = 4.9 Hz, 2H), 3.57 (t, J = 5.2 Hz, 2H), 3.46 (t, J = 6.1 Hz, 2H), 3.41–3.40 (m, 2H), 3.38–3.37 (m, 2H). HRMS (ESI): m/z calcd for C_40_H_32_N_8_O_5_Zn [M+H]^+^, 769.1860; found, 769.1847.

### 3.4. Preparation of CuI@PcNP and Control Formulations

The target nanoparticles (CuI@PcNP) and their corresponding controls (CuINP and PcNP) were fabricated via a facile nanoprecipitation method. Briefly, 1 mg of Cu_2_I_2_(PPh_3_)_2_(pz) was dissolved in 100 μL of DMSO with the assistance of heating and sonication to form the stock solution of the copper complex (10 mg/mL). 2.3 mg of Pc4OH was dissolved in 1000 μL of DMSO to a final concentration of 3 mmol/L. Separately, 5 mg of DSPE-PEG2K was dissolved in 100 μL of DMSO to create a lipid solution. Both solutions were filtered through a 0.22 μm syringe filter before use.

To formulate the organic phase for CuI@PcNP, the Cu-I cluster solution (100 μL, containing 1.0 mg of cluster), the DSPE-PEG2K solution (20 μL, containing 1.0 mg of polymer), and the Pc4OH solution (3.3 μL, containing 10 μmol of Pc4OH) were thoroughly mixed. Separately, 10 mL of ultrapure water was added to a 20 mL glass vial and stirred vigorously at 800 rpm at room temperature, serving as the aqueous phase. The prepared organic phase was then drawn into a syringe and injected dropwise into the aqueous phase. The mixture immediately turned into a colloidal dispersion, indicating nanoparticle formation. The colloidal dispersion was left to stir for an additional 30 min to ensure the complete diffusion of DMSO into the water and the stabilization of the nanoparticle structure.

To remove the organic solvent, unencapsulated small molecules, and excess DSPE-PEG2K, the as-prepared colloidal dispersion was purified and concentrated using an Amicon^®^ Ultra centrifugal filter unit (MWCO 30 kDa; Merck KGaA, Darmstadt, Germany). Specifically, the dispersion was washed three times with ultrapure water (5 mL each wash) by centrifugation at 2800× *g* for 10 min. Finally, the purified nanoparticles, designated as CuI@PcNP, were collected and resuspended in 2 mL of ultrapure water for storage at room temperature and subsequent characterization.

Two types of control formulations were prepared to serve as references in subsequent experiments. CuINP (nanoparticles containing only the Cu-I cluster) were prepared following the identical procedure for CuI@PcNP, but with the omission of the Pc4OH stock solution from the organic phase. PcNP (formulations containing only the photosensitizer) were prepared similarly, but with the omission of the Cu-I cluster stock solution.

The FRET efficiency was determined from the steady-state fluorescence of the donor. The calculation used the formula E = 1 − (F_DA_/F_D_), where F_D_ and F_DA_ represent the donor’s fluorescence intensities in the absence and presence of the acceptor, respectively. To isolate the donor’s signal, samples were excited at 475 nm, a wavelength where Pc4OH absorption is minimal. The donor’s fluorescence intensity was then measured at its emission maximum of 635 nm. Control experiments confirmed that the fluorescence emission from Pc4OH at 635 nm is negligible under these conditions, thus permitting an accurate measurement of F_DA_ without needing correction for spectral bleed-through. Spectra were recorded on an Edinburgh Instruments FS5 fluorimeter.

### 3.5. X-Ray Induced ROS Generation Assay

The X-ray-induced ROS generation was quantified using 2′,7′-dichlorodihydrofluorescein diacetate (DCFH-DA) as a fluorescent probe. Briefly, aqueous dispersions of CuI@PcNP, CuINP, and PcNP were incubated with a pre-hydrolyzed DCFH solution (final probe concentration: 10 μmol/L). The concentrations of the active components were maintained at 5 μmol/L for Pc4OH and 0.5 mg/mL for Cu_2_I_2_(PPh_3_)_2_(pz) in the respective nanoparticle groups. A DCFH solution in ultrapure water was used as a negative control. Upon exposure to X-ray irradiation (40 kV, 80 μA), the fluorescence intensity of the oxidized product, 2′,7′-dichlorofluorescein (DCF), was monitored over time at 525 nm (λₑₓ = 488 nm) to assess the rate of ROS production.

### 3.6. Cell Culture

Human hepatocellular carcinoma (HepG2) cells, obtained from the American Type Culture Collection (ATCC), were cultured in high-glucose Dulbecco’s Modified Eagle Medium (DMEM; Thermo Fisher Scientific, Waltham, MA, USA) supplemented with 10% fetal bovine serum (FBS) and 1% penicillin-streptomycin solution. The cells were maintained as an adherent monolayer culture at 37 °C in a humidified incubator containing 5% CO_2_.

#### 3.6.1. Cellular Uptake

The cellular uptake was quantified by flow cytometry [9]. HepG2 cells were seeded in 6-well plates at a density of 2 × 10^5^ cells per well and cultured overnight to allow for attachment. The culture medium was then replaced with fresh medium containing CuI@PcNP, CuINP, or PcNP (at an equivalent concentration of 5 μmol/L Pc4OH or 25 μg/mL Cu_2_I_2_(PPh_3_)_2_(pz)). After a 4 h incubation in the dark, the treatment medium was aspirated, and the cells were washed twice with cold phosphate-buffered saline (PBS) to remove non-internalized materials. Subsequently, the cells were detached using trypsin-EDTA, collected by centrifugation (1000 rpm for 3 min), and resuspended in PBS. The intracellular fluorescence intensity, originating from the Pc4OH component, was immediately analyzed using a flow cytometer to evaluate the cellular uptake efficiency.

#### 3.6.2. In Vitro Photocytotoxicity

The in vitro dark toxicity and X-ray-induced photodynamic cytotoxicity of the formulations were evaluated using the MTT assay. HepG2 cells were seeded into 96-well plates at a density of 1 × 10^4^ cells per well and incubated overnight. The cells were then treated with fresh medium containing serial dilutions of CuI@PcNP, CuINP, or PcNP for 4 h. Following incubation, the treatment medium was removed, and the cells were washed with PBS before adding 100 µL of fresh medium. For the photodynamic therapy group, the plates were immediately exposed to X-ray irradiation (40 kV, 80 µA) for 3 min. A parallel set of plates was kept in the dark to assess dark cytotoxicity. After treatment, all plates were returned to the incubator for an additional 24 h. Finally, cell viability was determined by a standard MTT protocol, with the absorbance of the resulting formazan solution measured at 492 nm using a microplate reader. Cell viability was expressed as a percentage relative to untreated control cells.

### 3.7. Animal Experiment

Female ICR mice (4–6 weeks old, 20–25 g) were purchased from Wushi Animal Co. Ltd. (Fuzhou, China). The subcutaneous H22 hepatoma tumor model was established by injecting 1 × 10^7^ H22 cells suspended in 100 μL of PBS into the right hind leg of each mouse. All animal studies were carried out in compliance with the guidelines of the Animal Ethics Committee of Fuzhou University (2023-SG-001), and were approved by the committee.

#### 3.7.1. In Vivo Fluorescence Imaging

Once the tumor volume reached approximately 200 mm^3^, the tumor-bearing ICR mice were used for the biodistribution study. The mice were intravenously administered via the tail vein with 100 μL of either CuI@PcNP (at a dose of 38 μg/kg Pc4OH and 2.5 mg/kg Cu_2_I_2_(PPh_3_)_2_(pz)) or PcNP (38 μg/kg Pc4OH). Whole-body fluorescence imaging was performed at various time points post-injection using an IVIS Lumina III imaging system (λ_ex_ = 605 nm, λ_em_ = 680 nm) to monitor the real-time distribution and tumor accumulation of the injected agents. The results revealed that the peak tumor fluorescence signal occurred at 12 h post-injection. At 28 h post-injection, mice (*n* = 3 per group) were euthanized, and major organs (heart, liver, spleen, lung, kidney) and tumors were excised for ex vivo imaging to confirm the biodistribution profile.

#### 3.7.2. In Vivo Photodynamic Anticancer Efficacy

The in vivo antitumor efficacy of the nanoparticles was evaluated using the H22 tumor-bearing ICR mouse model. When the tumor volume reached approximately 50–100 mm^3^, the mice were randomly allocated into six treatment groups (*n* = 5 per group): (1) PBS (Control); (2) PBS + X-ray; (3) CuINP; (4) CuINP + X-ray; (5) CuI@PcNP; and (6) CuI@PcNP + X-ray.

A single dose of the respective formulation (100 μL) was administered to each mouse via tail vein injection. The dosages were precisely controlled as follows: mice in the CuI@PcNP groups (5 and 6) received 38 μg/kg of Pc4OH and 2.5 mg/kg of Cu_2_I_2_(PPh_3_)_2_(pz); mice in the CuINP groups (3 and 4) received 2.5 mg/kg of Cu_2_I_2_(PPh_3_)_2_(pz).

Guided by the biodistribution study results, which indicated maximum tumor accumulation at 12 h post-injection, the designated groups (2, 4, and 6) were subjected to irradiation at this time point. The tumor region was exposed to X-rays (40 kV, 80 µA) for 8 min. To better simulate the X-ray attenuation effect in deep-seated tumors, a 5 cm thick slice of pork belly was placed between the X-ray source and the tumor during the procedure. The therapeutic outcome was assessed by monitoring and recording tumor volumes and body weights every other day over a 14-day period to evaluate both treatment efficacy and systemic toxicity. Tumor volumes were calculated using the formula: Volume = (length × width^2^)/2, and the results were expressed as relative tumor volume (V/V_0_).

## 4. Conclusions

In summary, we have developed a novel theranostic nanoplatform, CuI@PcNP, by co-encapsulating a discrete copper iodide cluster scintillator (Cu_2_I_2_(PPh_3_)_2_(pz)) and a phthalocyanine photosensitizer (Pc4OH) within a DSPE-PEG matrix. The rational design ensures the scintillator and photosensitizer are in close proximity, enabling highly efficient FRET (58%) upon X-ray excitation. This efficient energy transfer translated directly to superior therapeutic performance, with the CuI@PcNP system demonstrating enhanced ROS generation and significantly higher cytotoxicity against cancer cells in vitro. In vivo, the nanoparticles exhibited excellent tumor accumulation, enabling image-guided X-PDT to achieve a remarkable 77.4% tumor inhibition with excellent biocompatibility. This study validates the utility of Cu-I clusters as effective energy transducers in a DSPE-PEG-based formulation for X-PDT and provides a versatile strategy for developing synergistic, image-guided nanotherapeutics for deep-seated tumors.

## Data Availability

The data presented in this study are available on request from the corresponding authors.

## Supplementary Materials

The following supporting information can be downloaded at https://www.mdpi.com/article/10.3390/molecules30214229/s1, Figure S1: Schematic structure of Cu_2_I_2_(PPh_3_)_2_(pz). Figure S2: PXRD patterns of (a) Cu_2_I_2_(3-mpy)_4_, (b) Cu_2_I_2_(PPh_3_)_2_(3-mpy)_2_, and (c) Cu_2_I_2_(PPh_3_)_2_(pz). Figure S3: ^1^H NMR spectrum of PTOH in DMSO-*d*_6_. Figure S4: HRMS spectrum of PTOH. Figure S5: ^1^H NMR spectrum of Pc4OH in DMSO-*d*_6_. Figure S6: HRMS spectrum of Pc4OH. Figure S7: Solid-state photoluminescence spectra of the copper iodide clusters at room temperature. Figure S8: Spectral overlap between the photoluminescence spectrum of the energy donor Cu_2_I_2_(PPh_3_)_2_(pz) and the UV-vis absorption spectrum of the energy acceptor Pc4OH. Figure S9: Characterization of PcNP. Figure S10. UV-vis absorption spectra of (a) CuI@PcNP and (b) CuINP nanoparticles dispersed in water, monitored over a period of 3 days at room temperature. Figure S11: Fluorescence spectra of (a) CuI@PcNP and (b) CuINP nanoparticles dispersed in water, recorded over 3 days upon excitation at 360 nm. Figure S12: Digital photographs of tumors excised from H22 tumor-bearing mice on day 14 after various treatments. Scheme S1: Synthesis of the precursor PTOH and phthalocyanine derivative Pc4OH. Table S1. Photophysical properties of the synthesized copper iodide clusters.
