# Supplementary material for: Preparation and Performance of Phthalocyanine @ Copper Iodide Cluster Nanoparticles for X-Ray-Induced Photodynamic Therapy
Source: Molecules. 2025 Oct 29;30(21):4229. doi: 10.3390/molecules30214229 (PMC12610418; doi:10.3390/molecules30214229)
Supplement: Supplementary file 1 [file molecules-30-04229-s001.zip › molecules-3918505-supplementary.pdf]

Supplementary Materials

Preparation and Performance of Phthalocyanine @ Copper Iodide Cluster Nanoparticles for X-Ray-Induced Photodynamic Therapy

Wei Xie, Yunan Li, Guoyan Tang, Zhihua Li, Mengyu Yao, Biyuan Zheng, Xingshu Li * and Jian-Dong Huang *

Fujian Provincial Key Laboratory of Cancer Metastasis Chemoprevention and Chemotherapy,

College of Chemistry, Fuzhou University, Fuzhou 350108, China

*Address correspondence to Xingshu Li, xingshuli@fzu.edu.cn; Jian-Dong Huang, jdhuang@fzu.edu.cn

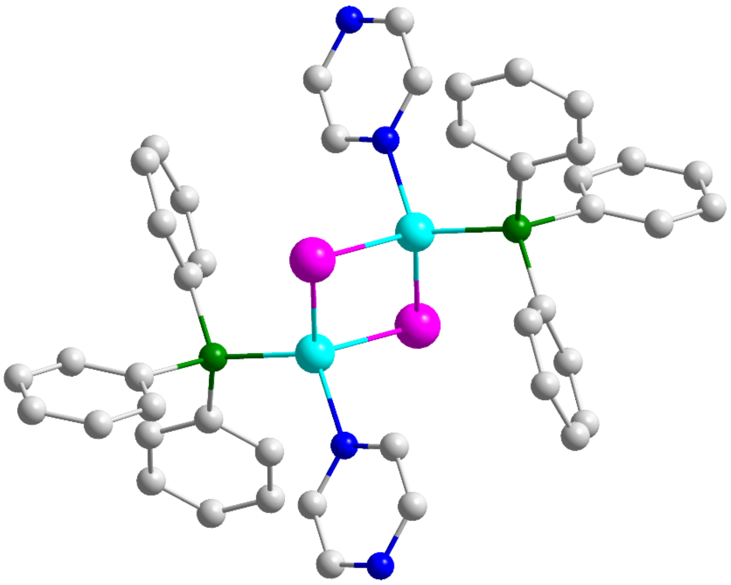

Figure S1. Schematic structure of $Cu_2I_2(PPh_3)_2(pz)$. Color code: Cu, cyan; I, magenta; P, green; N, blue; C, grey. Hydrogen atoms are omitted for clarity.

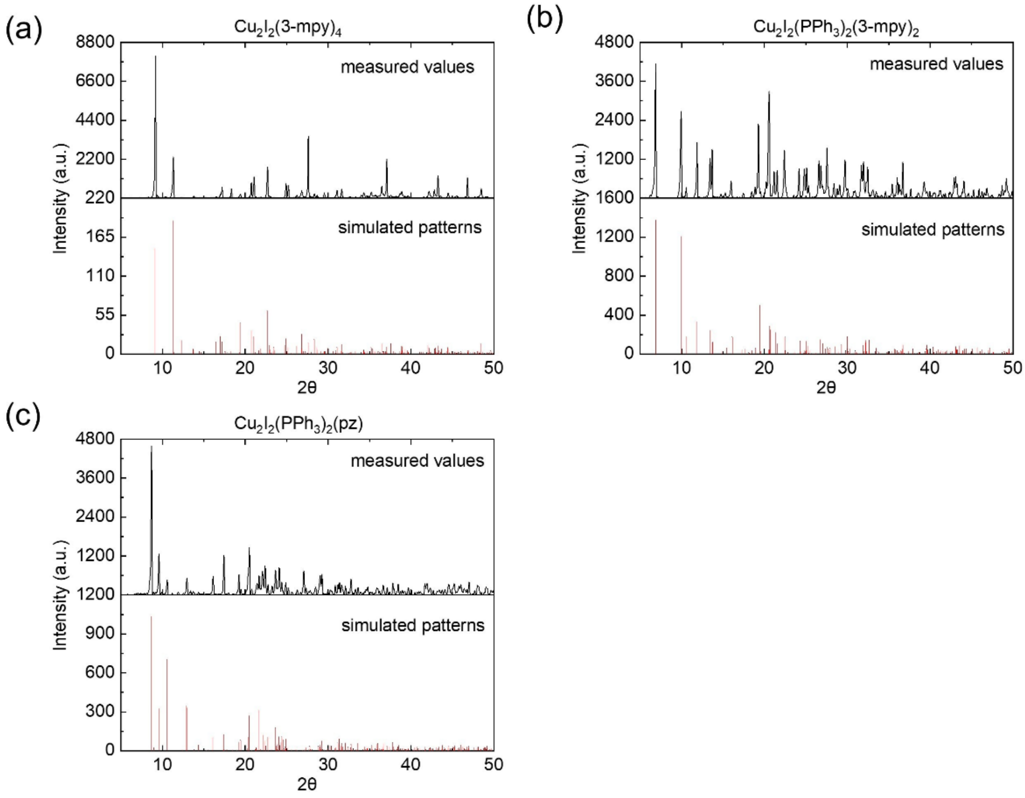

Figure S2. PXRD patterns of (a) $Cu_2I_2(3\text{-mpy})_4$, (b) $Cu_2I_2(PPh_3)_2(3\text{-mpy})_2$, and (c) $Cu_2I_2(PPh_3)_2(pz)$. The experimental patterns (black) are shown alongside the simulated patterns (red) for comparison.

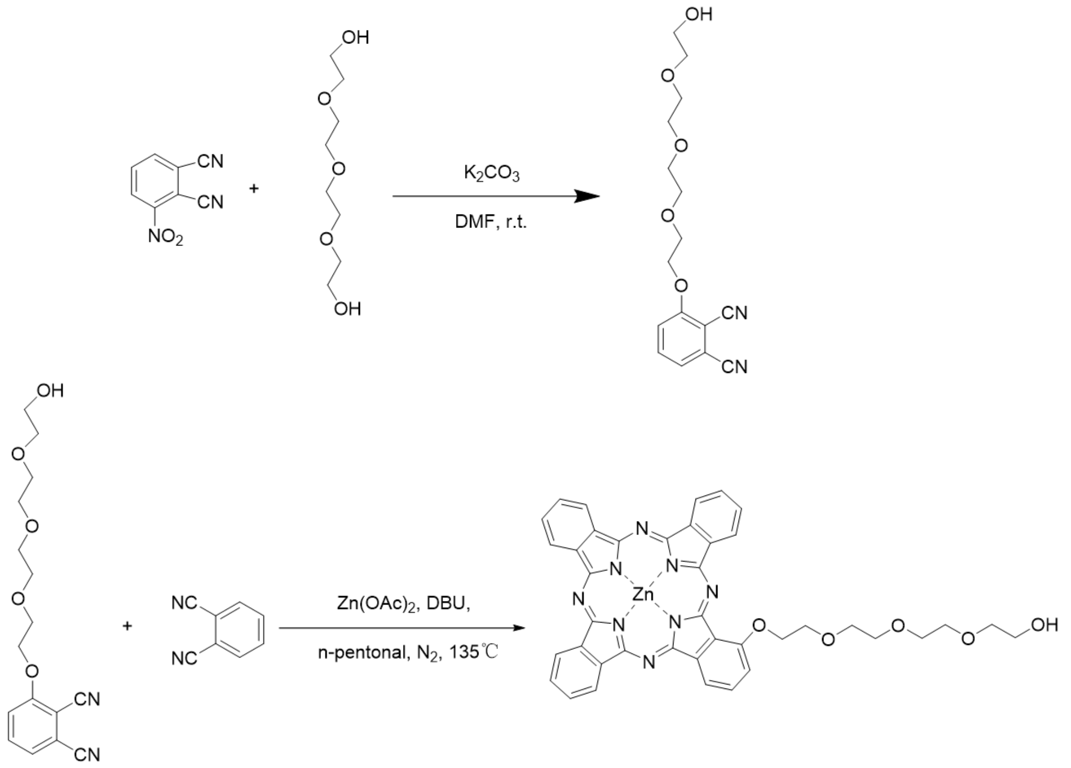

Scheme S1. Synthesis of the precursor PTOH and phthalocyanine derivative Pc4OH.

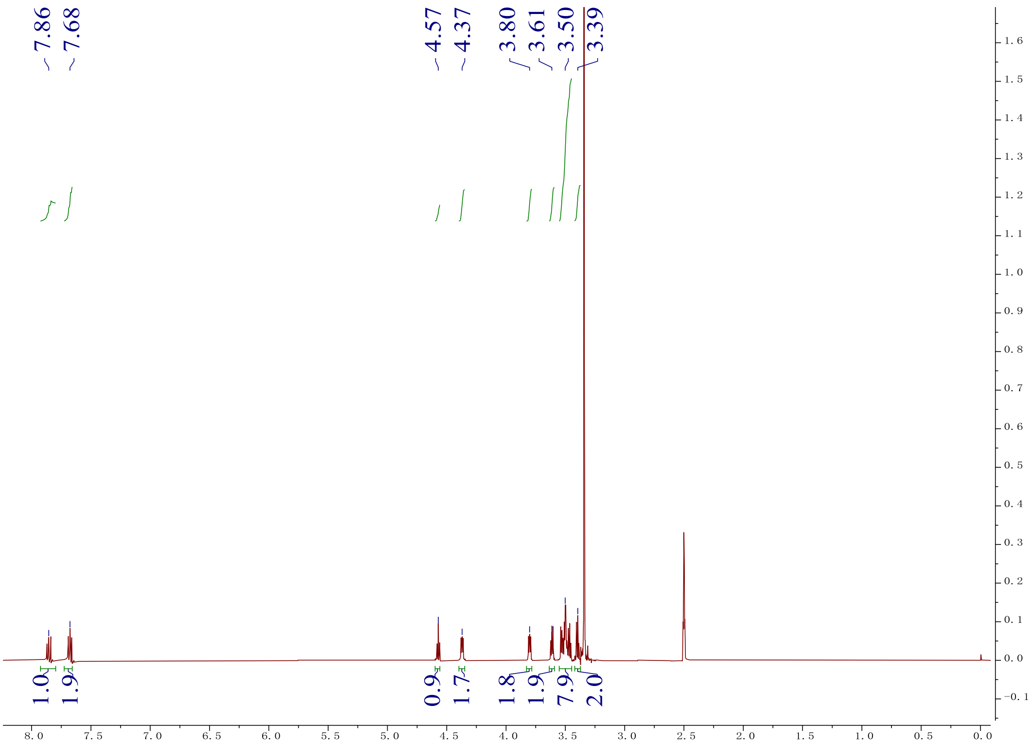

Figure S3. ^{1}H NMR spectrum of PTOH in DMSO-d_6.

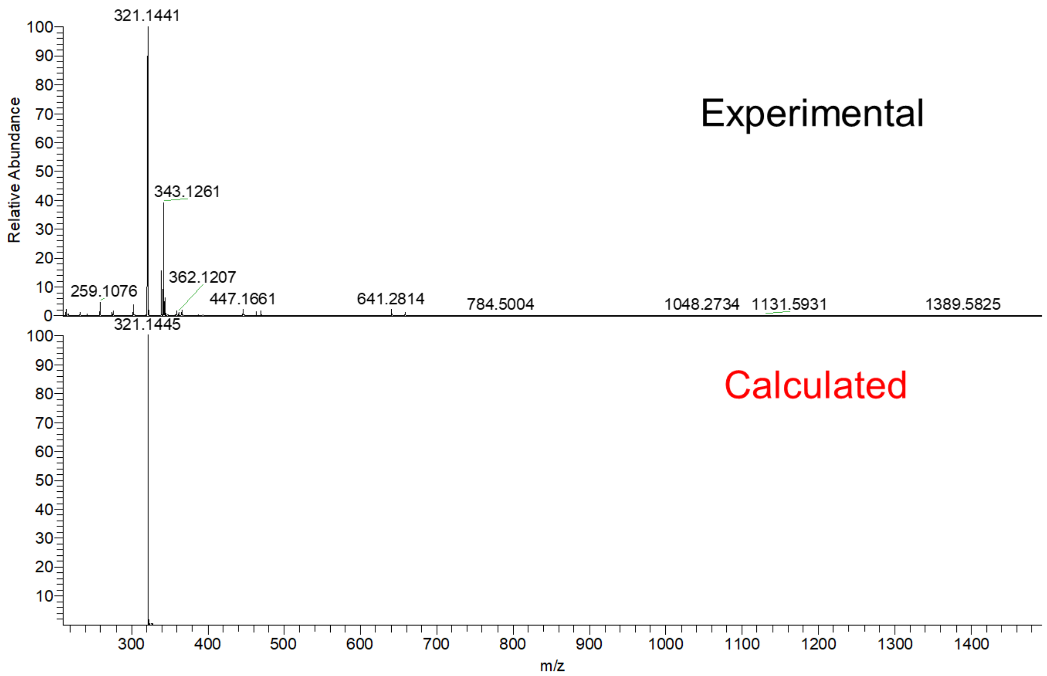

Figure S4. HRMS spectrum of PTOH.

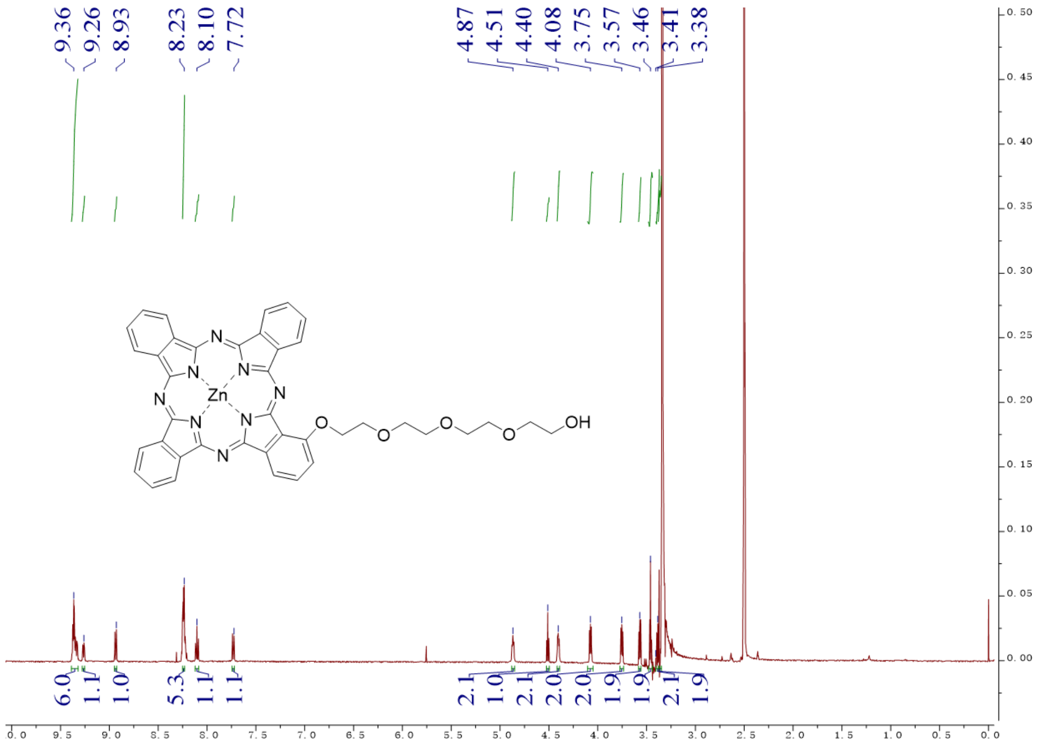

Figure S5. ^{1}H NMR spectrum of Pc4OH in DMSO-d_6.

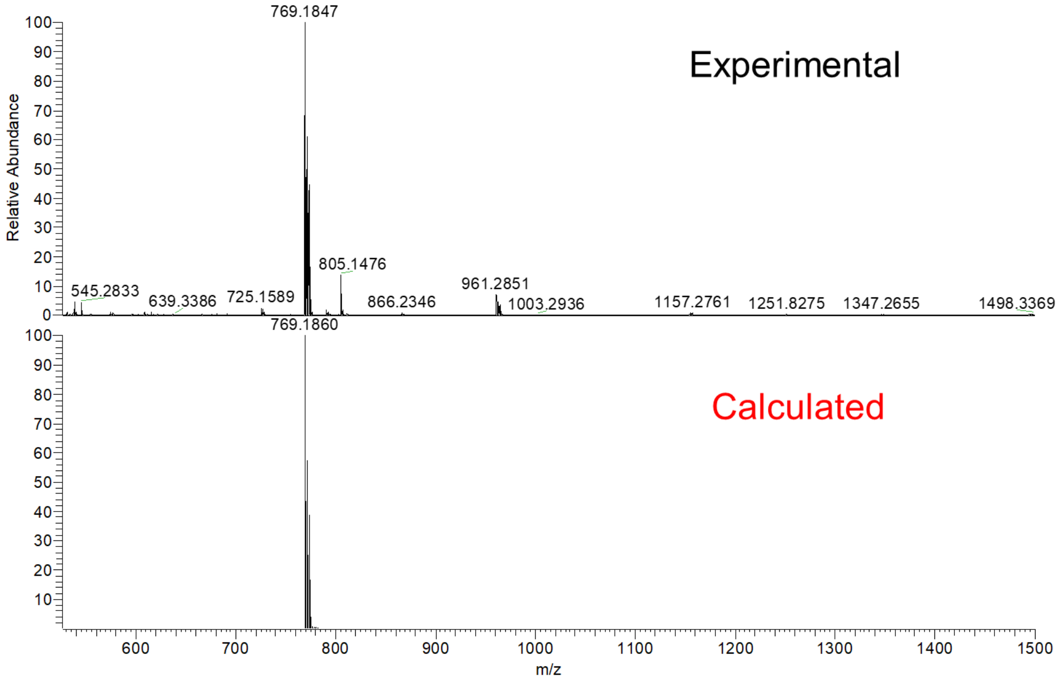

Figure S6. HRMS spectrum of Pc4OH.

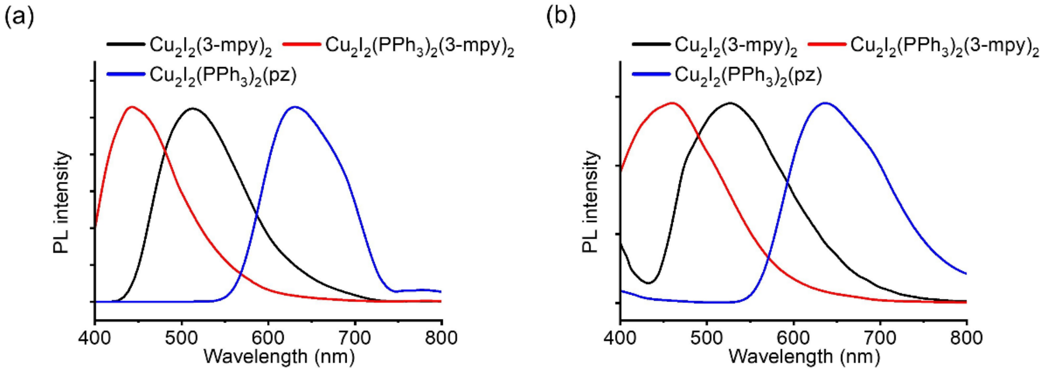

Figure S7. Solid-state photoluminescence spectra of the copper-iodide clusters at room temperature (a) X-ray excited luminescence (XEL) spectrum; (b) Photoluminescence spectrum under 360 nm excitation. All spectra are normalized for comparison.

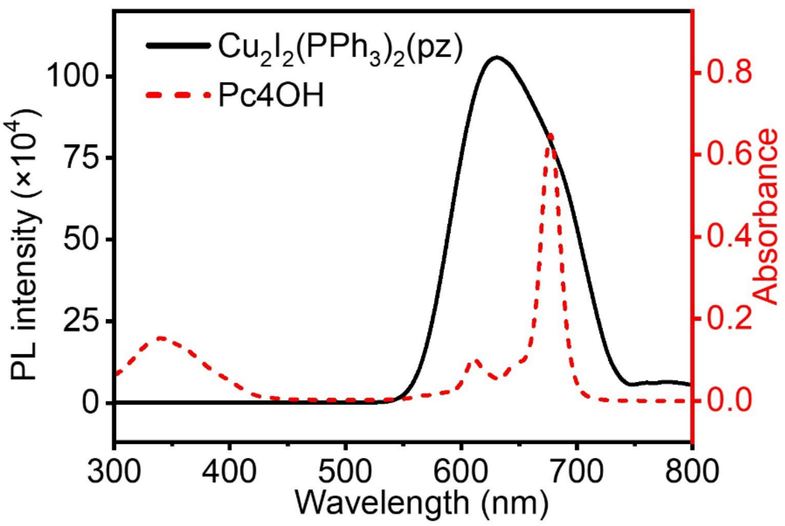

Figure S8. Spectral overlap between the photoluminescence spectrum of the energy donor $Cu_2I_2(PPh_3)_2(pz)$ (black solid line) and the UV-vis absorption spectrum of the energy acceptor Pc4OH (red dashed line).

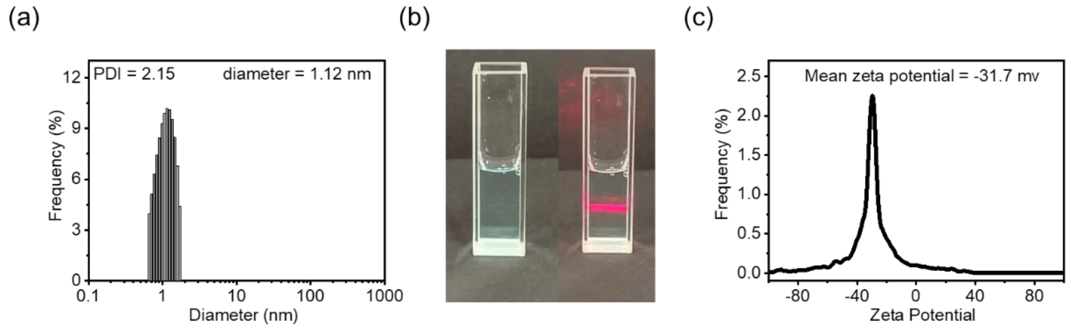

Figure S9. Characterization of PcNP. (a) Hydrodynamic size distribution of PcNP determined by DLS. (b) Photograph demonstrating the Tyndall effect of the PcNP solution. (c) Zeta potential measurement of PcNP.

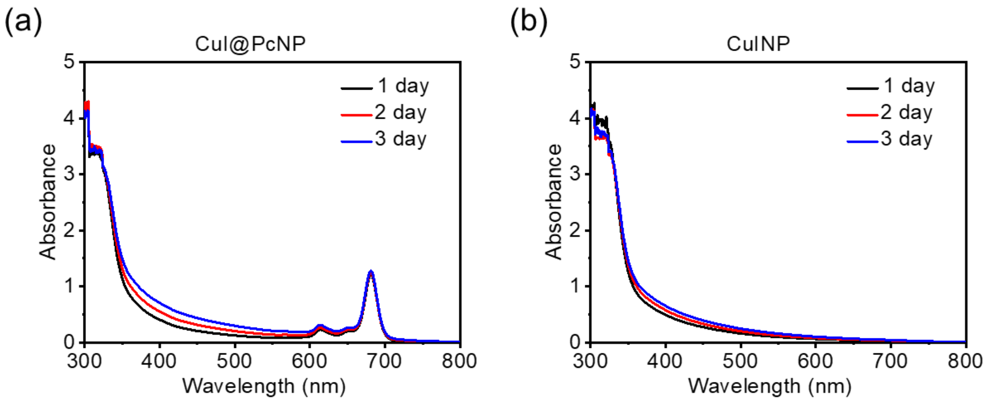

Figure S10. UV-vis absorption spectra of (a) CuI@PcNP and (b) CuINP nanoparticles dispersed in water, monitored over a period of 3 days at room temperature.

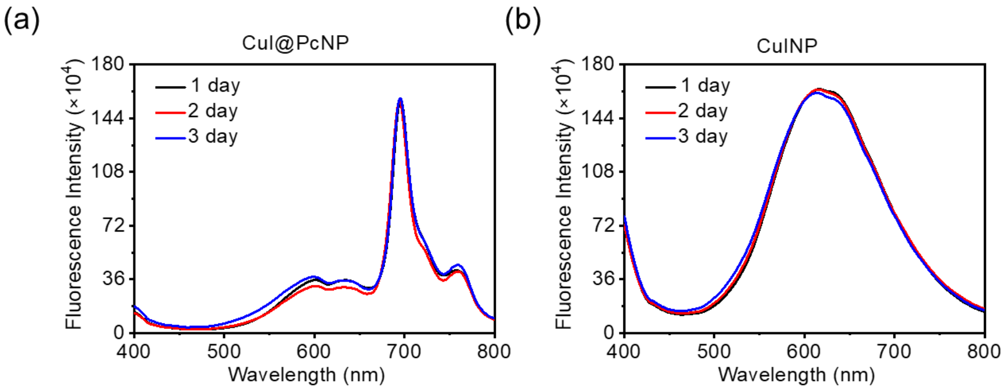

Figure S11. Fluorescence spectra of (a) CuI@PcNP and (b) CuINP nanoparticles dispersed in water, recorded over 3 days upon excitation at 360 nm.

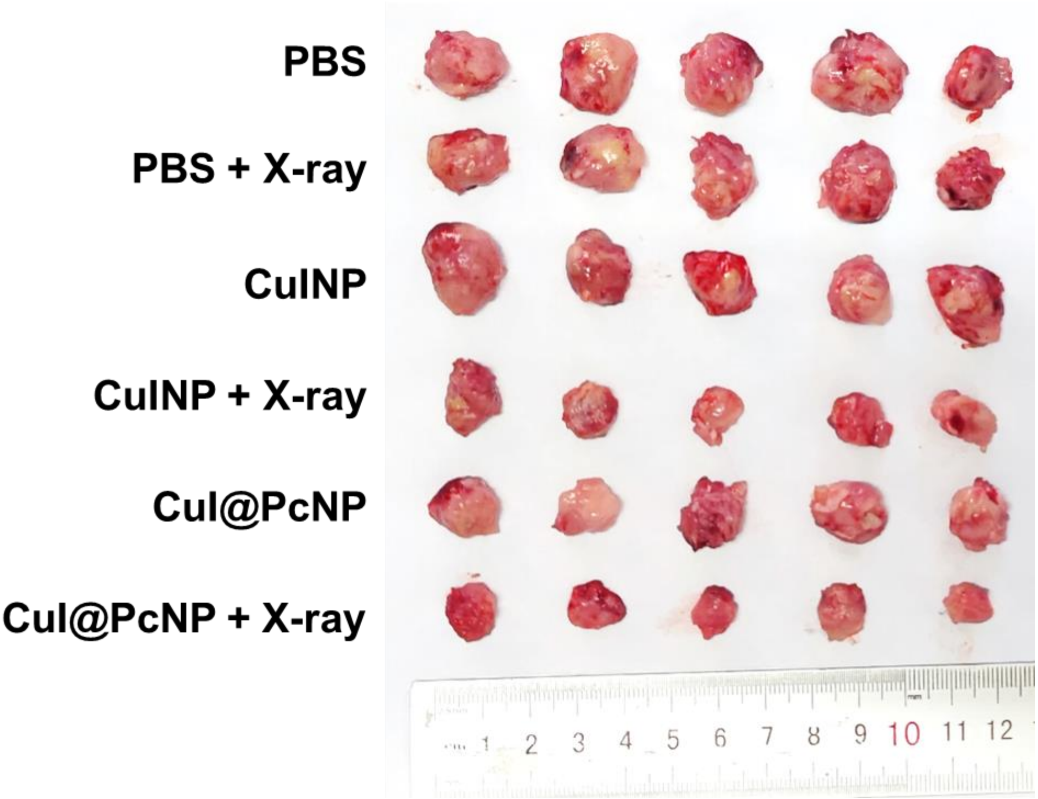

Figure S12. Digital photographs of tumors excised from H22 tumor-bearing mice on day 14 after various treatments. The treatment groups, from top to bottom, are as follows: (1) Saline, (2) Saline + X-ray, (3) CuINP, (4) CuINP + X-ray, (5) CuI@PcNP, and (6) CuI@PcNP + X-ray.

Table S1. Photophysical properties of the synthesized copper-iodide clusters.

Compound	$\lambda_{em, max}$ (X-ray ex.) (nm)	$\lambda_{em, max}$ (360 nm ex.) (nm)
$Cu_2I_2(3\text{-mpy})_2$	513	527
$Cu_2I_2(PPh_3)_2(3\text{-mpy})_2$	443	460
$Cu_2I_2(PPh_3)_2(pz)$	630	637
